# Collectanea

**Published:** 1832-01

**Authors:** 


### COLLECTANEA.

Floriferis ut apes in saltibus omnia libant,

Omnia nos, itidem, depascimur aurea dicta.

### PATHOLOGY.

*Hepatic and Splenic Derangement, simulating Organic Disease of the Heart or Aneurism of the Aorta.* By J. Franklin Vaughan, M.D., Physician to the Almshouse of Newcastle County, at Wilmington, Delaware.

A married lady, who had generally enjoyed good health, and was the mother of several fine children, was attacked, in the latter part of July 1825, with bilious fever, then prevalent in the district in which she resided. The attack being violent, and the fever of a highly inflammatory type, her physician (as he informed me,) had to resort to active and decisive measures for relief: depletion, free and repeated, by the lancet, active purging, and all the usual antiphlogistic means, were put in requisition. In the course of a short time the fever yielded, tonics were prescribed, the patient pronounced convalescent, and medical attendance discontinued.

In about six weeks, however, the doctor was summoned to visit his patient in haste, as she was "dangerously ill." He found her labouring under an urgent sense of suffocation, violent palpitation of, and a most distressing feeling of weight about the heart, with acute pain. Venesection was at once resorted to, and some relief afforded, but the pain in the region of the heart and the palpitation continued. A consultation with an aged and respectable practitioner was held, and her disease was pronounced to be either (*which*, they found it impossible to ascertain,) "an organic affection of the heart, or an aneurism of the aorta at its arch." Under these circumstances, a palliative course was adopted, consisting of venesection once or twice a week, according to the violence of the symptoms, with digitalis, absolute rest, low diet, anodynes at night, &c. This plan was continued for about two months, without any alleviation of the distressing symptoms; but, on the contrary, (as the patient stated,) they were all greatly aggravated, when I was called to see her.

Her situation at this time was truly deplorable: the palpitation of the heart was so violent as to throw up the bedclothes, at every diastole, so as to be distinctly seen across a large room! and the mental despondency was greater than I had ever seen it, despair seeming literally stamped upon the features. This state of mind, however, I was not much surprised at, when I learned from the lady that she had been made acquainted with the fearful diagnosis, and still more terrible prognostic, a sudden death. The patient was, as might be expected, very much debilitated; her skin was sallow and unhealthy; the bowels torpid, being evacuated only by medicine; and the pulse wiry or corded, quick and frequent.

After a minute inquiry into the history of the case, and a most careful examination of the thorax, both by mediate and immediate auscultation, I was induced to believe that there was really no primary or permanent disease of either heart or aorta. Some other cause for the situation of the patient was then to be sought for, and my attention was immediately directed (by the history of the case and the generally depraved state of the system,) to the chylopoietic apparatus: and here I soon found a state of things which confirmed the opinion already expressed, a very great enlargement and induration of the spleen; some enlargement, evident induration, and slight tenderness on pressure in the liver, and the usual debility and disorder of stomach consequent to biliary obstruction.

Might not then all her sufferings be referred to the derangement of the functions of these important organs, and all the indications of disease in the thorax be sympathetic and delusive?

In the subsequent consultation, this opinion was looked upon, by those in attendance previously, as fanciful or "speculative;" and the indications, or *methodus medendi* predicated upon it, positively condemned as being utterly inadequate to relieve the urgent and alarming symptoms, and only calculated to hasten the termination of an incurable disease! On, however, fairly, plainly, and explicitly stating the case in the presence of all concerned, my opinion, and the treatment founded upon it, were adopted, because it offered some hope, the other none.

The course pursued may be related in a few words: a very slight salivation was excited by the blue pill, and maintained for about four weeks; an epispastic applied over the liver and spleen, dressed, as soon as it could be borne, by mercurial ointment; a light but more nutritious diet was allowed, and cheerful society, &c. recommended. Soon after a gentle ptyalism was established, the obstructed secretions were restored, the torpid bowels became regular, and the enlargement of the liver and spleen were found to be yielding to it and the counter-irritants; a perceptible diminution in the violence of the palpitation, with relief of pain, weight, oppression, &c. directly followed, and the general health gradually and regularly improved, until a complete restoration was effected. During the convalescence, tonics (especially Cort. Peruv. with Rad. Valerian.) were freely administered, aided by a nutritious diet; and as soon as the strength was sufficiently increased, active exercise, especially riding, &c. Five years have now elapsed, and this lady continues perfectly well.

The preceding case suggests several remarks; but leaving others to draw their own inferences, I will only notice two, which appear to me of practical importance. In the first place, the only difficulty was in making a correct diagnosis, and yet the most efficient aid in forming it, auscultation, was entirely overlooked, and the idea that it really afforded any important information was treated with derision, and this by respectable, though certainly mere routine country practitioners! *Jam solvi nobile problema, dato aliquo morbo, invenire remedium?* Every one will admit the truth of this proposition; but the difficulty lies in the *dato morbo*, (the disease, not its name, being given,) which is, very frequently indeed, not the fact. The tissue affected, the nature and degree of its morbid lesion, or even the organ, or (more than this) even the cavity in which the disease is located, are often involved in great obscurity. I have always considered the diagnosis as the great difficulty in the practice of medicine, and therefore value above all others those works and those means which are calculated to throw any light upon a subject of such vital importance. Is it not, then, unpardonable in a practising physician to neglect any of those means, and especially when of such established utility as the stethoscope?

2d. The state of the pulse, and the appearance of the blood detracted in this case, are worthy of notice and comment: the former was frequent, corded, and tense, and the latter buffy and cupped, up to the period of my first visit; and these facts were confidently insisted on, not only in justification of the repeated bleedings, but as absolutely demanding their continuance. My views, however, were very different: I had seen this same synocula pulse, small, corded, frequent, and tense, exist in a state of alarming debility; had witnessed its removal by the use of tonics and a nourishing diet; and had been fully convinced that repeated bleeding might and would produce it, but never can, never did, remove it.

Accurately to discriminate between the irritable and the inflammatory pulse, though at all times of the utmost consequence, is occasionally exceedingly difficult. My excellent friend, and for some time preceptor, the distinguished

Dr. PARRISH, used to relate the following interesting example in his lectures. While Dr. WISTAR was in Edinburgh, he, with some other students, one day bled a dog to death. Just before he expired, a practitioner, who was well accustomed to feeling the pulse, happening to step in, was requested to place his hand on the dog's heart, (being unacquainted with what had been done,) and inform them whether the action was sthenic or asthenic? he made the desired examination, and pronounced the excitement to be sthenic!

This synocula pulse is frequently met with in the advanced stages of phthisis pulmonalis, accompanied by great prostration of strength; and also occurs occasionally in hectic fever, proceeding from diseased bones, old, indolent, and sloughing ulcers, where it is merely the effect of irritation; sometimes, indeed, it may be found just before dissolution, appearing to be only the last struggle of expiring nature.

Concerning the indications to be drawn from the appearances of the blood, Sir ASTLEY COOPER gives us a valuable lesson, or caution, in the following case: A man, in Guy's Hospital, in the last stage of scurvy, whose skin would ecchymose from the slightest pressure, and from whose gums blood was oozing, was bled, (a little being taken as an experiment,) and even here the blood was both sizy and cupped!—*American Journal of Med. Sciences.*

---

*Account, by a Physician, of the successful Treatment of Empyema by Paracentesis in his own Person. (Journal der Praktischen Heilkunde.)*

IN the year 1810, and the twenty-third year of his age, Dr. Wendelstadt, who had commenced the practice of his profession a year previously, was attacked with pleurisy, which he did not pay particular attention to, but treated as an attack of rheumatism. By and by he was annoyed by a constant sense of heat and oppression in the right side, where the inflammation had existed: he was unable to lie on the left side for difficulty of breathing; and frequently in the night time was, for the same reason, obliged to resume the sitting posture. He had at the same time constant pain at the point of the shoulder, soreness in the windpipe and larynx, convulsive cough with difficult mucous expectoration, and a slow fever, which gradually put on the form of hectic. Notwithstanding his incessant sufferings, however, he recovered strength so far in the course of six months that he was able laboriously to follow his profession. Not long afterwards he consulted a medical friend, who found the ribs of the right side protruded, the whole right side motionless on respiration, but the intercostal spaces not protruded. Those who question the utility of the stethoscope and the pathological researches of Laennec, will perhaps acknowledge that something at least has been added to the certainty and facility of our diagnosis in diseases of the chest, when we state that in the year 1811 Dr. Wendelstadt's friend inferred from the symptoms that his disease was either thickening of the pleura and adhesion of the lungs to the parietes, or effusion. Various remedies were fruitlessly tried; but the Doctor continued to practice his profession, custom having rendered his sufferings supportable. Meanwhile the circumference of the chest continued to increase; climbing produced excessive dyspnœa; at length the presence of a fluid in the chest became evident by the sense of fluctuation, which was produced whenever the chest was agitated, and this was remarked first in the right side only, but afterwards as the enlargement increased, in the left also. About this time he was much annoyed by a singular affection, an uneasy feeling in the affected side when he went into warm air, which seemed to him to arise from the fluid within the chest not coming to an equilibrium of temperature with the surrounding medium so quickly as the parietes did. An operation was now proposed as the only means of relieving him; but he confesses that the accounts of recorded cases were not very encouraging; and therefore he continued to toil at his professional employment and to struggle with his misfortunes, "being supported," says he, "by his originally strong constitution, his cheerful temper of mind, his happy matrimonial connexions, the consciousness of doing his duty to the best of his ability, and resignation to his fate."

At last, in June 1817, he sustained a fresh attack of pleurisy, which terminated in increase of the effusion and difficulty of breathing, with extreme wasting, delirium, and other symptoms of an approaching termination to his sufferings, when he resolved to submit to the operation of paracentesis, in the hope of at least preserving himself a little longer for his family's sake. This was seven years after his disease began. The incision was made between the sixth and seventh ribs, when a milky-coloured fluid rushed forciby from the tube of the trochar. When half a quart was discharged the opening was closed up, after which he felt prodigiously relieved, slept soundly and awoke refreshed and lively. Next day a third of a quart was twice withdrawn; and on the third day as much more. He thinks, however, that the fluid was withdrawn in too large quantity; for he was immediately seized with impending suffocation, and continued in that state for twelve hours, the nails of his fingers being all the time livid, and his strength completely exhausted. Diffusible stimulants roused him from this alarming state; but it was followed by an insidious fever, œdema of the limbs, and painful hemorrhoids. On the fourth day the fluid was thicker in consistence, and fetid, and it continued more or less fetid for a fortnight. After that it was allowed to flow at each dressing as far as it would; and it became thick, white, and viscous. The fluctuation in the right side had ceased. It was now agreed to try the effect of gently astringent injections, together with the introduction of tents; but in six weeks no change of consequence was effected on the state of the chest. The œdema, however, was carried off by an incidental diarrhœa; in eight weeks he was able to go about the house, and in four weeks more to see his friends. The injections were then abandoned as useless and annoying, the injections of oak-bark decoction, in particular, having always excited rigor of several hours' duration, followed by heat of skin, quick pulse, and thirst, and finally by a sweating stage. In no long time he resumed his professional occupations, though still extremely feeble, and constantly exposed to fresh accessions of pain and fever from accidental exposure to cold.

In this manner he has now lived thirteen years; during which time the fluid has been daily withdrawn twice, sometimes half a drachm only issuing each time, sometimes so much as three or four ounces daily. During that period he has sometimes passed an entire half year without any uneasy feeling in his chest, or any other complaint but general weakness; but he has had frequent attacks of pain in the chest and feverishness, attended with great discharge. Three years ago he could not help gratifying his curiosity to know how large the cavity in his chest was, and on injecting warm water he found it held a quart without his feeling any inconvenience. He has never had any irritation in the lungs since the operation, although he is convinced there is a communication between the right lung and the cavity, because, when he draws in his breath and squeezes, as if at stool, he distinctly hears a hissing or whistling within the chest; and if he does this while in the bath, bubbles of air escape from the aperture, which he can keep up as long he pleases.

His present state is as follows, in the author's own words. "My complexion is fresh, my eye lively, my muscles weak, but my body not thinner than when I enjoyed perfect health in my early years. My appetite, digestion, and sleep are natural. Deep inspiration, a forced cough, or lying on the right side, excites pain in the aperture, and I lie best on the left side or on the back. The right side of the chest does not move at all when I breathe; yet I can blow the flute as well as another, and can walk faster than many people who are in perfect health. The right side is bent inwards, the right shoulder depressed, and the whole right side of the body weaker and less muscular than the left: I have no cough. Every time the dressing is changed I can, by quick contraction of the chest, blow out air from the aperture with such force as to extinguish a candle held at the distance of four or six inches. The discharge amounts to two ounces per day; it is purulent, brick-coloured, with occasional streaks and clots of blood, and it has the odour of milk. Fourteen days ago, an exfoliated piece of rib, half an inch long and two lines broad, came away by the aperture. after some previous feverishness and pain in the neighbourhood. As to my state of mind, my long years of suffering have, I think, weakened my memory; but my judgment, serenity of disposition, and self-possession, when beside my patients, have sustained no diminution.—*Edinburgh Med. and Surg. Journal.*

---

### PRACTICAL MEDICINE.

*Cases of Tic Douloureux, in which the Cyanuret of Potassium has been employed with Success.* By M. le Docteur LOMBARD, of Geneva.

THE cyanuret is employed in friction: it is either dissolved in distilled water, or made into an ointment with pure hogs-lard, and used in either form, according to circumstances. From one to four grains in an ounce of water is the quantity usually employed for the aqueous solution: from two to four grains in an ounce of lard is the composition of the ointment. It may be mentioned that the aqueous solution is in general, of the two forms, the more prompt and instantaneous in its effects.

CASE I. *Facial Neuralgia instantaneously cured by the Hydrocyanate of Potash in Friction.* A lady, of robust habit, forty-nine years of age, was a martyr to the most agonizing occasional attacks of pain in the space between the temporal region and the ciliary arch and maxilla. She used to scream violently in these torturing accessions, and sometimes lost all appearance of sensibility to such a degree, that she has been supposed to be struck with apoplexy. Pulse eighty-four; face rather flushed; no functional derangement. She was ordered to be rubbed with the aqueous solution, containing sixteen grains of cyanuret of potassium in four ounces of distilled water: it was rubbed on the forehead and cheek with a ball of cotton. The pain gave way almost instantaneously at the very first application; and seemed, as the patient said, to be rubbed away with the hand. A complete cure was effected by persevering a little while in the use of the remedy.

CASE II. *Periodic Neuralgia removed by the Ointment of the Cyanuret.* The cure in this instance was less prompt, but not less certain. A lady, thirty-eight years of age, experienced the severest pains in the temporal region and upper jaw of the left side; they came on regularly every morning at four o'clock; went on increasing in severity until about ten, and did not cease till four in the afternoon. In that interval she laboured under anorexia, fever, headach, &c. and was almost driven distracted. She was bled to twelve ounces to relieve congestion, and then the ointment was applied to the cheek and temple. Two grains to the half ounce of lard were at first employed, but the improvement was more rapid under the application of ten grains to two ounces. Lotions of the cyanuret were eventually used, and the cure was complete.

CASE III. *Facial Neuralgia almost immediately cured.* A lady, twenty years of age, suffered for several days, at the same hour, the most torturing pains in the orbital and supra-maxillary region. Her face was much flushed, particularly on the affected side. Ten grains of the cyanuret were dissolved in four ounces of distilled water, and rubbed on with cotton. The application was quite successful.

CASE IV. *Chronic Occasional Neuralgia similarly treated.* A woman, of eighty, who had long suffered from irregular attacks of this complaint, was cured by lotions and frictions, compounded pretty strongly, and continued for some time.

The cyanuret of potassium is contra-indicated where the nervous affection is complicated with inflammatory action, discharge, &c. It is a useful remedy in non-inflammatory rheumatism.

In sciatic neuralgia it has not been successful; nay, it has been necessarily discontinued, on account of some unpleasant accidents which it occasioned.

In white swelling, attended with acute pain, poultices moistened with the solution had the effect of producing much comfort, though the continuance of their application had no promise of amendment in it.

It is on the whole inferred that the calming properties of this remedy are superior to those of any other known, and that it should always have a preference where inflammation does not exist. Lotions, with hydrocyanic acid, are by no means to be compared with it, for the acid is decomposed with facility, and scarcely to be used without danger.

The first application of the cyanuret in the above way is claimed by M. Buttigny and his brethren of Geneva, but it is disputed with them by Messrs. Robiquet, Villaumé, and Bally, of Paris.—*Gazette des Hôpitaux; Med. Gazette.*

---

### SURGERY.

*Case of Inguinal and Popliteal Aneurism, cured by tying the External Iliac Artery.* By Alexander Ewing, m.d., Surgeon to the Royal Infirmary, and Lecturer on Surgery to the Universities of Aberdeen.

Robert Petrie, thirty-seven years of age, of a sallow complexion and general unhealthy appearance, was admitted into the Aberdeen Infirmary, on the 22d December, 1830, with aneurism of the popliteal artery of the right side, which he said had been of several weeks' duration. He had been a sailor since he was fifteen years of age, and for ten years had made voyages to the West Indies, where he had several very bad fevers, and narrowly escaped with his life. By the account of his friends, he had also been much addicted to drinking spirits.

The tumor in the ham was large, and, from the swelling of the integuments, appeared diffused, filling the space between the hamstring muscles, and extending upwards on the inside of the thigh, to near where the femoral artery pierces the triceps; the leg was swollen and a little red, particularly about the upper part. He complained of excruciating pain about the knee and down the outside of the leg, which prevented him from sleeping, particularly during the eight days previous to admission. He said the tumor was of seven weeks' duration, and that he knew no cause for it, except that, a week before observing it, he fell into the forecastle of a vessel, a height of about five feet, and at this time he felt a sharp pain in the ham.

When he first discovered the tumor, it was about the size of a nut, and for a considerable time it pulsated more strongly than when he came to the hospital. Pulse about eighty; tongue a little furred; appetite impaired; bowels slow. On examining the thigh more particularly after admission, with a view to tying the femoral artery, another tumor was discovered in the situation of that vessel, just after emerging under Poupart's ligament. This was of the size of a walnut, and had not been noticed by the patient for more than a fortnight. By grasping it with the finger and thumb placed on opposite sides, it was felt to be strongly distended at each pulsation of the artery; and, by pressure on the external iliac above the crural arch, the pulsation ceased, and the tumor almost disappeared.

It appeared, therefore, that the patient had two aneurisms, an inguinal and popliteal, on the same side. As this seemed to indicate some predisposition in the vascular system, I examined the abdomen, chest, and pulse, in order to ascertain if there were any internal aneurism; but nothing of this kind could be detected. He informed me, however, that he had a rupture on the same side when young, and that it was cured by a bandage.

During the time he was in the hospital previous to the operation, he only got opiates to allay pain and procure sleep, and occasional laxatives.

On consulting about the case, it was agreed that the only chance which the man had was by tying the external iliac; although from his bad constitution, deteriorated by warm climates, former diseases, and intemperate habits, and the probable diseased state of the arteries, an operation did not hold out much prospect of success.

As my friend Mr. Liston, of Edinburgh, happened to pass through the town at the time, he had the kindness to examine the case, and concurred in the opinion that the external iliac should be tied. I accordingly performed the operation on that day, (3d January, 1831,) with the assistance of Mr. Liston and my colleagues. The first incision commenced as high as the anterior and superior spinous process of the ilium, but an inch nearer the linea alba. This was carried down in a somewhat semilunar direction, and terminated a little above the external aperture of the inguinal canal. The tendon of the external oblique muscle was then divided on a director to the same extent, bringing into view the internal oblique. The struggles of the patient were very violent after this, and, by forcing down the abdominal viscera, made it necessary to proceed with great caution. The next steps of the operation consisted in pinching up the muscular fibres of the internal oblique with a dissecting forceps, and carefully dividing them in the direction of the external wound, and in the same way some of the fibres of the transversalis. The fascia transversalis being then exposed, a few fibres of it were carefully scratched through with the knife, on the iliac side of the exit of the cord through the internal aperture of the inguinal canal. The peritoneum was then pushed upwards and inwards, bringing into view the external iliac, about an inch and a half above Poupart's ligament. The cellular tissue surrounding the artery and vein was next scratched through by a few touches of the knife on the upper surface of the artery, and a single ligature was then passed under it from within outwards, by the common aneurism needle. A firm double knot was tied, and both ends of the ligature were left uncut and hanging from the wound. The pulsation in both aneurisms immediately ceased. It may be observed that no part of the artery was separated from its connexions, except in the tract of the needle. The operation was concluded by bringing the edges of the incision together by three stitches, some strips of adhesive plaster, a light compress, and putting a broad flannel roller round the loins and upper part of the thigh.

For an hour after the operation, the leg was quite cold and extremely painful. During this time external warmth was applied; but in the course of the evening the temperature rose two degrees higher than that of the opposite limb, and the pain at the same time diminished.

For the first two days he was troubled with sickness and vomiting, and slept little, but the pulse continued about eighty. During this time he got castor oil to open his bowels, and saline effervescing draughts, with small doses of opium. His bowels were freely opened on the first day after the operation, but the sickness and retching continued. He was then allowed soda water to drink, which had the effect of settling his stomach. In a few days some inflammation appeared about the wound, and the stitches were cut out. This was followed by suppuration, but adhesion soon took place, except at a small spot round the ligature. No pulsation ever returned in the popliteal aneurism after the operation; but there was a slight pulsation in the one at the groin, which at the same time felt solid, as if filled with a coagulum. About a fortnight after the operation, this patient was accidentally exposed to the contagion of smallpox, by a boy who took it after coming to the hospital. As Petrie had never been vaccinated, he was immediately ordered to be inoculated, and he went through the regular stages of cowpox, without any apparent bad effect on the wound or otherwise.

It is unnecessary to give details of the further progress of the case, except to state that the patient's health continued good, and the aneurismal tumor in the ham gradually diminished. The wound healed up rapidly, but the ligature did not come away till the 7th March, probably in part from my unwillingness to use any force to pull it away, while the granulations firmly embraced it.

The patient, after sitting up for some time in the hospital, walked home perfectly cured; and I saw him some days ago in good health.—*Edinburgh Med. and Surg. Journal.*

---

*Nitric Acid in Toothach*, by Dr. Ryan. Since my former note upon the extraordinary success of this acid, in giving immediate relief, when properly and cautiously applied to caries of the teeth, I have used it in many cases with invariable success. It should be applied with a gold or glass probe covered with lint, as a silver probe decomposes the acid, and renders it ineffectual. It is therefore necessary to cover the ordinary probes with lint very lightly, and to apply the acid quickly to every part of the carious surface. If the disease ascends high into the fang by a fine opening, complete relief cannot be obtained, unless the extremity of the nerve is touched, and this is a difficulty which is often met with, when the upper teeth are affected. In general, the application affords immediate relief without the slightest pain.—*London Med. and Surg. Journal.*

---

### MIDWIFERY.

*A Case of Anomalous Labour*, by Thomas Ferguson, m.d. I am induced to lay the following rare and embarrassing case before my medical brethren, from a conviction that recording such unusual deviations of Nature from her ordinary course must have a beneficial tendency, in apprising the junior practitioner of the many trying and arduous situations he may expect to be placed in, during the course of his professional career, and in some degree prepare him to discharge his duty to a confiding public with fidelity, with credit to himself, and honour to the profession of which he is a member.

On the 19th of June, 1829, I was taken out four miles from town to see a lady who had been in strong labour from five o'clock the preceding evening. On my arrival at 11 a.m. I found in attendance the family physician, and a gentleman whom I understood to be a practitioner in midwifery; as also a midwife, who had had charge of the case from its commencement.

The report I received was, that early the preceding evening the midwife supposed the presentation to be the head and one hand, but finding the labour making little or no progress, she expressed her fears to the husband of the lady, that other aid might become necessary; on which the medical gentlemen already mentioned were summoned. I was further informed by one gentleman, that the pains continued severe through the night, with very short intervals, and that not until morning was it discovered that the feet were presenting; on which my assistance was sought for.

I proceeded to make an examination, and found the feet protruding almost without the os externum; the toes pointing to the perineum; the os uteri fully dilated, and the soft parts in an advantageous state to facilitate delivery. The urinary bladder was much distended, and a profuse perspiration covered the entire body of the patient. Her mind was exceedingly agitated by alarming forebodings of painful operations, ultimate danger, &c. I endeavoured to assuage the apprehensions of the lady, and also of her husband, by an assurance that the case was neither unusual nor formidable, which I hoped soon to demonstrate satisfactorily.

By desire of the medical gentlemen, I proceeded to the delivery, and having previously emptied the bladder of its contents, co-operating with the pains, I found no unusual obstacle in the progress of the labour, until the child's body was so far protruded as to enable me to ascertain, by the pulsations of the funis, then without the os externum, that the child was alive. From this stage of the delivery I began to experience a most unusual, and to me unaccountable resistance to the further descent of the child, and, notwithstanding the aid of powerful labour-pains and considerable manual exertions, I found great difficulty in advancing the body so low as to extricate and bring down the arms, the trunk still remaining so high that the hands and forearms only were without the os externum. Every subsequent effort at delivery I found to be unavailing.

On further examination, I discovered there were twins, and, to my great embarrassment, ascertained that the head of a second child had entered the pelvis, while, by passing my finger along the spine and neck of the child then partly delivered, I could distinctly trace its head still remaining above the brim of the pelvis. My first efforts were to endeavour to push up the head of the second child then descending, so as to allow the other head, that of the first child, to occupy its proper place. But all my exertions proved ineffectual, owing to the powerful contractions of the uterus, which had been much facilitated in pressing the second head into the pelvis by the removal of the obstruction, which it before met in the arms of the first child: indeed it would appear that this obstruction had retarded the descent of the second head at a much earlier period of the labour.

Finding there was no possibility of extracting the poor little sufferer from his then perilous situation, and perceiving the pulsations in the funis still strong, I resolved in my own mind immediately to employ the perforator on the head of the second child then within my reach. But, to crown my difficulties, I found myself without the means, having left town without instruments. Thus situated, I informed my patient's husband of the perplexing nature of the case, and despatched one of the medical gentlemen to a very contiguous village to procure instruments. Fortunately the mission was attended with considerable delay, and ultimately failed.

During the time thus spent, notwithstanding all my care to preserve warmth in the child partly born, the circulation in the funis became languid, and at length extinct. Nevertheless I was agreeably consoled by the progress made in the descent of the head of the second child being so far advanced as to give reasonable hopes that nature would prevail, and accomplish her work, which she actually effected in the birth of one living child: (the second, in which the head presented,) which was accompanied by the expulsion of the head of the other, still-born, two hours and a half after the arms had been extracted as described above.

Before I attempt a description of this interesting process of parturition, I should premise that it was the lady's second confinement, that she appeared to possess great fortitude of mind, was remarkably well formed, and had throughout powerful uterine action. The child which first presented, a male, was of such a size that in the beginning I had not the slightest suspicion of twins; the second, a female, was not so large.

The process of parturition was as follows. The descent of the footling was in the most favorable position for delivery, the toes pointing to the sacrum, and must have been progressing in that regular course, until interrupted by the intrusion of the second head into the pelvis, so soon as the arms and shoulders of the first had fairly cleared its brim. Here then the second head must have made its way into the pelvis; its right ear to the sacrum, its left to the pubes. At this time the neck of the first child occupied the left angle of the pelvis, posterior to the head of the second, the head resting on the brim of the pelvis, ready to follow the descent of its companion; its left ear turned to the pubes, the right to the sacrum. In the labour process the head of the male child became imbedded in the hollow of the neck of the female, its left ear occupying a situation nearly approaching the right ear of the female, but a little higher up. The frontal bone of the female must have rested on the clavicle and shoulder of the male child, as was quite evident from the effects of the uterine action; for in proportion as the head of the female advanced, so did the arms and shoulders of the male clear the os externum.

The face of the female when entirely protruded was turned upwards, the mother then lying on the left side. At this time the male head remained within the vagina, and it was only during the violent pains, which protruded the shoulders of the female, that the head of the male fell out on its face in the bed. The delivery was soon completed by the natural expulsion of the secundines.

The abdomen remained much distended after delivery. The following day it became tender on pressure, and, more immediately in the region of the uterus, considerable pain was complained of; but the curative means usually had recourse to on similar occasions being energetically employed, these unfavorable symptoms gradually subsided, and my patient recovered so as to be enabled to nurse her daughter.

Three cases somewhat analogous, but differing widely in their peculiar circumstances, more especially in the treatment adopted and their results, may be found in the Medico-Chirurgical Transactions of London, vol. 12, p. 336, to which I beg leave to refer the reader.—*Dublin Medical Transactions.*

---

### MISCELLANEOUS.

*Galvanism.* The most formidable battery of large plates that has hitherto been constructed, is that described by Mr. J. G. Children, in the Philosophical Transactions for 1815. The plates were two feet six inches wide, and six feet high, the copper being opposed to both surfaces of the zinc. These enormous plates were properly fastened to a beam of wood, suspended by counterpoises from the ceiling of the laboratory, so as to be readily and safely immersed into or removed from the cells of acid, which were twenty-one in number, and their united capacities amounted to 945 gallons. A leaden pipe, three fourths of an inch diameter, was attached to the extreme plate at either end, and immersed into separate basins of mercury, by means of which perfect metallic contact was ensured. The charge consisted of a mixture of nitric and sulphuric acids, with thirty, or occasionally only twenty, parts of water. When the poles of this arrangement were united by a platinum wire, eleven hundredths of an inch in diameter, it became redhot for a length of five feet six inches. In the same way it ignited eight feet six inches of the same wire, of forty-four hundredths of an inch; and a bar of platinum, one sixth of an inch square, and two and a quarter long, was not only heated to bright redness, but fused at the end.—*Elements of Chemistry.*

---

*Mortality of Leeches during Storms.* That atmospheric changes have a remarkable influence upon leeches is a well established fact. In 1825, M. Derheims, of St. Omer, ascribed their very sudden death, at the approach of or during storms, to the coagulation of the blood of those creatures, caused by the impression of the atmospheric electricity. This opinion, which at that time was the result of theory, he has since confirmed by direct experiments. —*Philosoph. Journal.*

